# The cutting‐edge progress in bioprinting for biomedicine: principles, applications, and future perspectives

**DOI:** 10.1002/mco2.753

**Published:** 2024-09-23

**Authors:** Shuge Liu, Yating Chen, Zhiyao Wang, Minggao Liu, Yundi Zhao, Yushuo Tan, Zhan Qu, Liping Du, Chunsheng Wu

**Affiliations:** ^1^ Department of Biophysics Institute of Medical Engineering School of Basic Medical Sciences Health Science Center Xi'an Jiaotong University Xi'an Shaanxi China; ^2^ Key Laboratory of Environment and Genes Related to Diseases (Xi'an Jiaotong University) Ministry of Education of China Xi'an Shaanxi China

**Keywords:** bioprinting, biosensor, disease modeling, microfluidics, regenerative medicine

## Abstract

Bioprinting is a highly promising application area of additive manufacturing technology that has been widely used in various fields, including tissue engineering, drug screening, organ regeneration, and biosensing. Its primary goal is to produce biomedical products such as artificial implant scaffolds, tissues and organs, and medical assistive devices through software‐layered discrete and numerical control molding. Despite its immense potential, bioprinting technology still faces several challenges. It requires concerted efforts from researchers, engineers, regulatory bodies, and industry stakeholders are principal to overcome these challenges and unlock the full potential of bioprinting. This review systematically discusses bioprinting principles, applications, and future perspectives while also providing a topical overview of research progress in bioprinting over the past two decades. The most recent advancements in bioprinting are comprehensively reviewed here. First, printing techniques and methods are summarized along with advancements related to bioinks and supporting structures. Second, interesting and representative cases regarding the applications of bioprinting in tissue engineering, drug screening, organ regeneration, and biosensing are introduced in detail. Finally, the remaining challenges and suggestions for future directions of bioprinting technology are proposed and discussed.

Bioprinting is one of the most promising application areas of additive manufacturing technology that has been widely used in various fields. It aims to produce biomedical products such as artificial implant scaffolds, tissues and organs, and medical assistive devices. This review systematically discusses bioprinting principles, applications, and future perspectives, which provides a topical description of the research progress of bioprinting.

## Graphical abstract

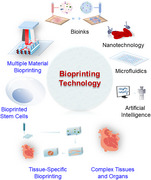

## 1 INTRODUCTION

**FIGURE 1:**
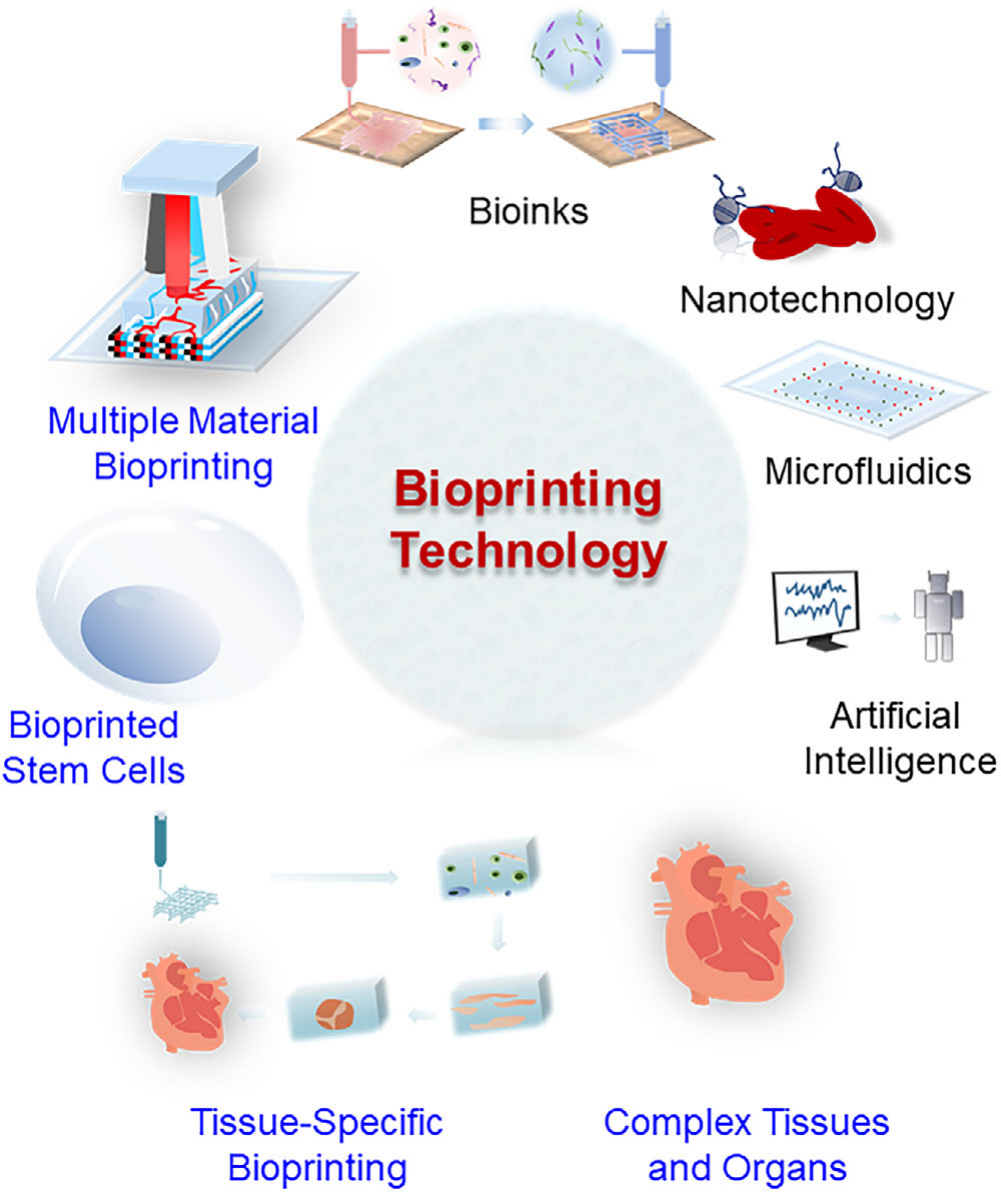
Most recent advances in bioprinting technology and its applications.

Bioprinting is a groundbreaking technology that is based on three‐dimensional (3D) printing, enabling the precise fabrication of complex biological structures by layer‐by‐layer deposition of bioinks. In recent years, a number of researches in bioprinting has gained significant momentum, with several notable advancements as summarized in Figure 1. Bioinks are the basis of bioprinting. Quite a few researchers have made progress in developing bioinks with improved printability, biocompatibility, and biofunctionality. These bioinks often incorporate natural materials, such as alginate, gelatin, or chitosan, as well as synthetic polymers Gelatin Methacryloyl (GelMA),^1^ to achieve desired mechanical and biological properties. Complex tissues and organs are one of the most promising applications of bioprinting. Cases of bioprinted complex tissues and organs, such as liver,^2^ heart,^3^ and blood vessels^4^ have been developed successfully. These achievements have involved the precise deposition of multiple cell types and the incorporation of vascular networks within the bioprinted constructs. Integrated with other technologies makes bioprinting more powerful. Microfluidics,^5^ nanotechnology,^6^ and artificial intelligence^7^ have been used to enhance the functionality and complexity of bioprinted tissues. Tissue‐specific bioprinting has also shown promising prospects. To replicate the unique microenvironments and cellular arrangements found in different tissues, researchers have focused on developing tissue‐specific bioprinting techniques, tailoring the printing parameters and bioinks to specific tissue types.^8^, ^9^ Bioprinted stem cells have also made significant progress in recent decades. The integration of stem cells is related to tissue regeneration capabilities. The use of stem cells in bioprinting allows for the differentiation of cells into specific lineages, leading to the formation of functional and mature tissues.^10^ Multiple material bioprinting is the key to current bioprinting technology. Simultaneous deposition of different biomaterials and cells could be realized by multimaterial bioprinting.^11^ This approach facilitates the creation of complex structures with spatial control over cell distribution and biomaterial composition.

Naturally, there are numerous advantages to bioprinting. One of the crucial benefits of bioprinting is its ability to precisely control the deposition of biomaterials in 3D space. Bioprinting techniques allow for the precise placement of multiple components, enabling the creation of complex tissue architectures with controlled spatial organization.^12^ This level of precision and control is vital for mimicking the native tissue microenvironment and promoting cell viability, proliferation, and differentiation. Bioprinting offers unparalleled customizability, allowing for the fabrication of tissues and organs that are tailored to individual patients’ specific needs.^13^ By manipulating the printing parameters, such as cell types, biomaterials, and structural design, bioprinting enables the creation of constructs with desired mechanical properties, biochemical cues, and vascular networks.^14^ This customizability allows for the development of patient‐specific tissue models, personalized medicine approaches, and the potential for organ transplantation without the need for immunosuppression. Importantly, bioprinting provides a reproducible and standardized approach to tissue modeling. By utilizing computer‐aided design (CAD) software and robotic systems, bioprinting ensures consistent and precise deposition of cells and biomaterials, resulting in reproducible tissue constructs.^15^ This reproducibility is essential for drug screening assays, where consistent and reliable tissue models are required to assess drug efficacy, toxicity, and pharmacokinetics.^16^ Meanwhile, bioprinting provides in vitro models that closely mimic human physiology, reducing the reliance on animal testing for drug screening and toxicity studies as well as accelerating research and development.^17^ Most importantly, bioprinting holds the potential to address the organ shortage by producing functional organs for transplantation, reducing the dependency on donor organs.

While bioprinting technology holds great promise, it also faces certain limitations and challenges that need to be addressed for its widespread application and commercialization. For example, bioprinting processes can impact cell viability and functionality, requiring further optimization to ensure optimal cellular behavior and long‐term functionality of printed constructs.^18^ Another challenge is the creation of functional vascular networks within bioprinted tissues, as the intricate branching and perfusion required for large‐scale tissues are difficult to achieve.^4^ Moreover, the choice of suitable biomaterials for bioprinting is critical. Biomaterials must possess appropriate mechanical properties such as certain viscosity and gelation capability,^19^ biocompatibility, biodegradability, and bioactivity to support cell growth, differentiation, and tissue regeneration,^20^, ^21^ However, the availability of biomaterials that meet all these criteria is still limited. Developing advanced biomaterials that closely mimic the native tissue microenvironment and provide optimal support for cellular activities remains an ongoing challenge. While bioprinting can create tissue‐like structures, ensuring proper cell maturation, tissue integration, and functional outcomes is still a long way to go to enhance the long‐term viability and functionality of bioprinted tissues. Bioprinting complex tissues and organs often requires the integration of multiple cell types with specific spatial arrangements. Achieving the precise positioning and functionality of different cell populations within a bioprinted construct remains a problem. Strategies to optimize cell viability, functionality, and interactions are necessary to ensure the successful integration of multiple cell types in bioprinted tissues. Besides, scalability, production costs, and ethical regulation of large‐scale tissues or organs for clinical applications remain a major challenge.

In summary, although bioprinting technology has demonstrated significant potential, it still faces several challenges. Overcoming the scalability barrier, achieving vascularization, optimizing biomaterial selection, addressing regulatory and ethical considerations, promoting long‐term functionality, reducing production costs, integrating multiple cell types, and establishing standardization are all critical areas of focus for the future advancement and widespread adoption of bioprinting technology.^22^ Collaborations among researchers, engineers, regulatory bodies, and industry stakeholders are essential to overcome these challenges and unlock the full potential of bioprinting. This review comprehensively summarizes and discusses the most recent progress in bioprinting with regard to printing techniques and methods as well as advancements related to bioinks and supporting structures. Additionally, detailed examples of applications of bioprinting in tissue engineering, drug screening, organ regeneration, and biosensing are provided. The remaining challenges and suggestions for future directions of bioprinting technology are also proposed and discussed. Furthermore, bioprinting has been applied in other cutting‐edge research areas such as organoids, full‐scale human organs, and bioprinting under extreme conditions. However, due to space limitations, these areas will not be covered in detail here.

## 2 PRINCIPLES AND METHODS OF BIOPRINTING

**FIGURE 2:**
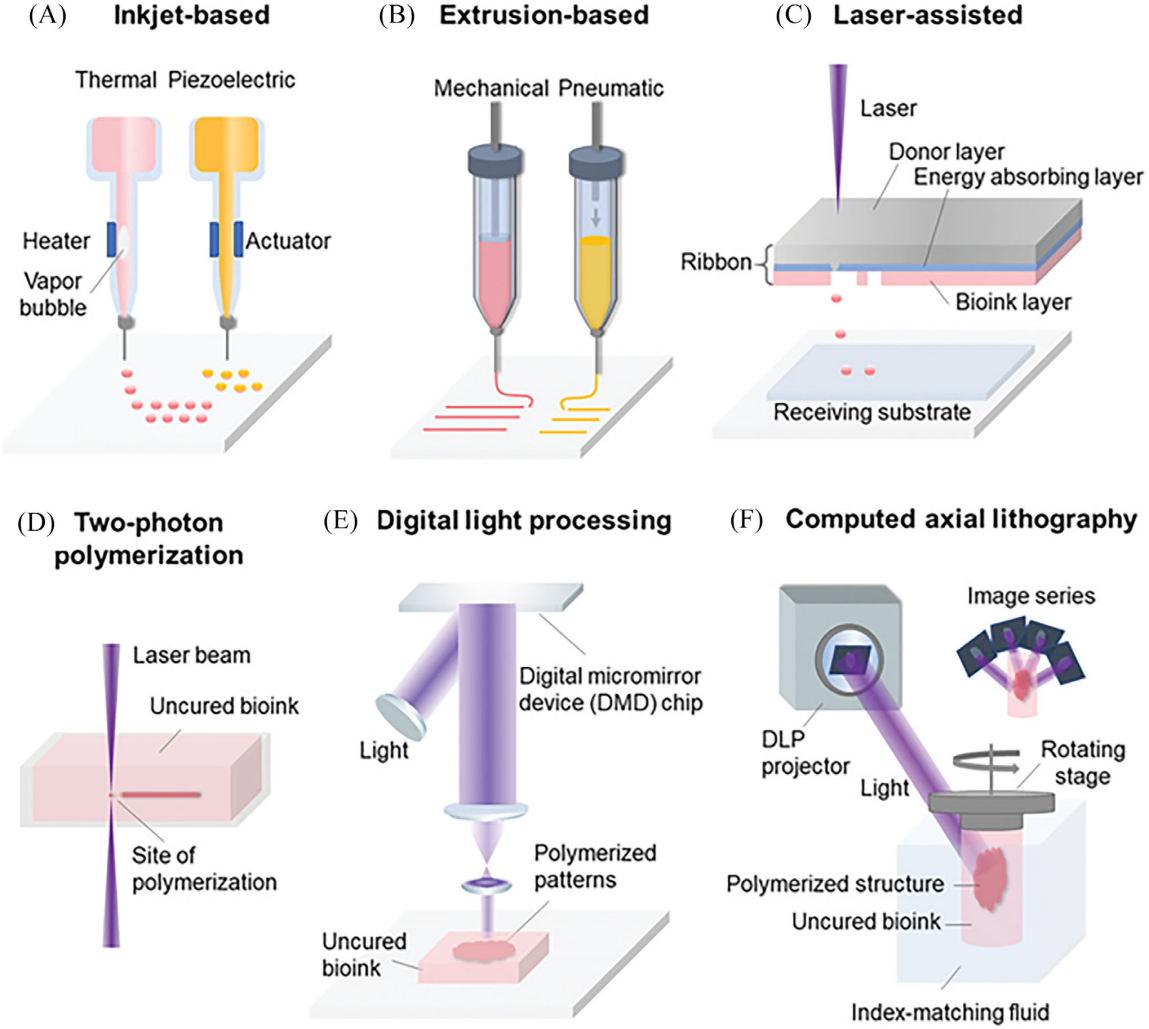
Schematic illustrations of 3D bioprinting technologies.^200^ Copyright 2020, John Wiley and Sons. (A) Inkjet‐based bioprinting. (B) Extrusion‐based bioprinting. (C) Laser‐assisted bioprinting. (D) Two‐photon polymerization‐based bioprinting. (E) Digital light processing‐based bioprinting. (F) Computed axial lithography.

Printing techniques, bioinks, supporting materials, and sacrificial structures are fundamental principles underlying bioprinting.^23^ First of all, various printing techniques are employed in bioprinting (Table 1). Inkjet bioprinting is the earliest biological 3D printing technology. It utilizes thermal or piezoelectric printheads to generate droplets of bioink that are ejected onto a substrate (Figure 2A). Bioinks are loaded into ink cartridges, and precise droplets are ejected onto target locations. This technique allows for high‐resolution and rapid printing speeds, precise control over droplet size and placement and the ability to pattern different cell types or biomaterials at the microscale.^24^ However, high viscosity materials and high concentrations of cells cannot be printed because of the low driving pressure of the nozzles. The low viscosity material makes the structural strength smaller after printing and molding, which cannot meet the demand of subsequent in vitro culture and transplantation, so the viscosity factor makes the range of applicable biomaterials narrow.^25^ In addition, mechanical or thermal damage to cells may occur during inkjet printing, and these drawbacks also limit the wide application of inkjet printing technology.^26^ Extrusion‐based bioprinting technology (Figure 2B) is currently the most widely used bioprinting method, and its greatest advantage lies in the wide range of biocompatible materials that can be printed (including cell clusters, cell‐carrying hydrogels, microcarriers, and decellularized matrix components, etc.), which covers biomaterials with viscosities ranging from 30 mPa/s to 6 × 10^7^ mPa/s.^27^ Commonly used extrusion‐based bioprinting methods include electric,^28^ pneumatic,^29^ piston‐driven,^30^ and screw‐driven^31^ systems. In addition, some researchers have begun experimenting with light‐curing technology combined with extrusion bioprinting to achieve higher print resolution and accuracy.^32^ It can also be tightly integrated with multimaterial, coaxial bioprinting for the fields of tissue engineering and regenerative medicine. However, the main limitation is the potential damage to cells during the extrusion process, as high shear stress could affect cell viability.^33^ Light projection bioprinting refers to the on‐demand projection of a light source into a precursor solution of light‐curing material, which is combined with three‐dimensional motion to achieve on‐demand curing of the solution in the cylinder and to obtain the corresponding three‐dimensional structure. Laser bioprinting technology (Figure 2C), which uses laser pulses to generate pressure waves to eject biological ink droplets onto a receiving substrate, offers a high‐resolution printing method.^34^ This technology has a wide range of applications and can be used to print a variety of biological materials, such as cells, proteins, and nanoparticles. But cell viability can also be affected by laser‐induced stress^35^ or metal nanoparticles formed during the laser bioprinting process.^36^ Stereolithography (SLA)^37^ uses point‐projection polymerization to induce the polymerization of photosensitive materials by precisely controlling the laser scanning cross‐section profile through a rotating mirror, in conjunction with continuous scanning, to form a three‐dimensional entity layer by layer. The printing method offers high accuracy and resolution due to the precise movement and tiny size of the laser,^38^ but single‐beam printing can also be more time consuming. In addition, the researchers used near‐infrared femtosecond laser‐induced two‐photon polymerization (Figure 2D) for point‐projection printing, which allows for the fabrication of ultrafine three‐dimensional structures on the nanoscale. Digital light processing (DLP) (Figure 2E) systems use an array of digital micromirrors to control the intensity of light at each pixel and to generate a surface‐projected light source, which polymerizes the photosensitive polymer material within the area of exposure to the projected light. The total print time for surface projection polymerization depends on the thickness of the structure and can be effectively reduced for printing large and complex structures. Computerized axial lithography (CAL) (Figure 2F) tomography technique polymerizes 3D structures by iteratively optimizing the azimuthal superposition of light projections through time‐multiplexed exposures. Moreover, CAL enables the construction of smooth surfaces with the potential to fabricate most tissues and organs.^39^ CAL's fabrication speed is also at the forefront of all 3D printing technologies, significantly accelerating the process from experimental to clinical. CAL‐based volumetric additive manufacturing technology is a major advancement in additive manufacturing due to its unprecedented manufacturing speed and resolution.^40^ Although CAL can theoretically achieve high resolution, it has three problems similar to those of traditional DLP methods^39^: (1) The technique is only compatible with photosensitive materials, which limits its ability to fabricate components containing multiple materials or microstructures. (2) The effect of oxygen content attenuation and diffusion of oxygen or inhibited molecules on the accuracy of the technique needs to be further investigated. (3) Scattering and superposition of light can affect fabrication accuracy. But, with improved algorithms and in‐depth analysis of light, the technique will likely lead to technological breakthroughs, especially in the fields of bio‐3D printing and regenerative medicine. If the technology can be more adapted to the real needs of biomedical applications, it will provide a transformative tool for tissue engineering and regenerative medicine.

**TABLE 1:** Advantages, disadvantages, and main technical parameters of different printing technologies.

Item	Advantages	Disadvantages	Printing speed	Resolution/µm	Costs	Applicable materials	Applications
Inkjet bioprinting^41^	High print speed, wide availability, uniform droplet size and controlled spray direction, open‐pool nozzle‐less ejection system, multiple cell and material types	Thermal and mechanical stress damage, and unreliable cell encapsulation, cell membrane damage and cleavage, limited material viscosity (ideally below 10 centipoise)	75 mm/s	100–500	Low	Bioinks (cell suspensions or gels containing cells)	Cell culture, tissue repair, fabrication of complex tissue structures, drug screening, etc.
Extrusion‐based bioprinting^42^	Multirange of materials viscosities, deposit very high cell densities	Cell viability is lower than that with inkjet‐based bioprinting, low printing speed and resolution	10∼50 μm/s	100–500	Low	Bioinks (gels containing cells, e.g. gelatin, alginate, etc.)	Tissue engineering, organ building, artificial skin manufacturing, drug screening, etc.
Laser bioprinting^43^, ^44^	Nozzle‐free transfer of material allows for a wide range of bioinks; absorbing layer minimizes laser effects	Slow printing; lower mechanical strength of materials; difficulty printing	1.6 μm/s	20–100	Medium	Photosensitive polymers, bioinks (gels containing cells)	Tissue construction, cell culture, biomedical research, etc.
Stereolithography^45^	Fast printing speed; high mechanical strength	Photoinitiator cytotoxicity; limited printable materials	10.5 cm/s	10–100	High	Photosensitive polymers, bioinks, bio‐based polymers	Tissue engineering, bionic organ manufacturing, cell culture, drug screening, etc.
Two‐photon polymerization^46^	Very high resolution	Very slow printing speed, limited volume, limited printable materials	7 mm/s	0.1–5	Medium	Photosensitive polymers, bioinks, bio‐based polymers	Microtissue structure fabrication, bionic organ fabrication, biomedical research, etc.
Digital light processing^47^, ^48^	Very fast printing speed	Photoinitiator cytotoxicity; limited printable materials; limited control on layer thickness	33 mm/s	10–100	Medium	Photosensitive polymers, bioinks, bio‐based polymers	Tissue engineering, organ building, bionic organ manufacturing, biomedical research, etc.
Computerized axial lithography^48^	Highly accurate, material diversity, flexible print shapes	Long manufacturing time, material residue	20 mm tall 3D geometry formed in less than 1 min	Submillimeter	High	Photosensitive polymers, bio‐based polymers, composites	Tissue engineering, organ building, bionic organ manufacturing in the biomedical field.

Second, bioinks are crucial components in bioprinting that provide necessary support and a nutritionally suitable microenvironment for cell growth and tissue formation. Commonly used bioink materials are hydrogels, including alginate, gelatin, collagen, and fibrin, which provide a suitable 3D matrix for cell encapsulation and proliferation.^49^ Other bioink materials, such as decellularized extracellular matrix^50^ and synthetic polymers including agarose,^51^ poly(ethylene glycol)^52^ and polyurethane,^53^ are also being explored for their ability to mimic the native tissue microenvironment. The selection of bioinks depends on many factors, including biocompatibility, printability, mechanical properties, and cell interaction. In recent years, several research teams have begun to explore the use of degradable materials as an option for bioinks.^54^, ^55^ Such materials are able to degrade gradually in vivo and provide a better biocompatible environment.

The last thing is supporting materials and sacrificial structures. It provides mechanical support during printing and facilitates the formation of complex tissue architectures. Supporting materials, such as hydrogel scaffolds^56^ or biocompatible polymers,^57^ help maintain the structural integrity of the designed construct and provide mechanical stability of the whole system. Sacrificial structures, on the other hand, are temporary structures that can be removed after bioprinting to create hollow channels or vascular networks within tissue construct. The sacrificial materials used for 3D printing sacrificial templates are categorized into hydrogel and polymer. 3D printing sacrificial hydrogel includes alginate,^58^ gelatin,^59^ agarose,^60^ and customized hydrogels such as hyaluronic acid (HA)‐based templates consisting of the guest–host pairs of adamantane (Ad) HA (Ad–HA) and β‐cyclodextrin (β‐CD) HA (CD–HA),^61^ xanthan gum in hydrogel matrices,^62^ or pH‐sensitive water‐soluble shellac in the hydrogel.^63^ With the advancement of sacrifice‐based techniques, several polymers and synthetic materials have also been explored to fabricate engineered vasculature. Among them, polyvinyl alcohol,^64^ polylactic acid,^65^ pluronic F‐127,^66^ poly(N‐isopropylacrylamide),^67^ saccharide,^68^ and polycaprolactone^69^ are the most common sacrificial materials. These sacrificial structures can be developed from materials that can be easily dissolved or extracted, such as sugar‐based materials^70^ or thermally responsive polymers.^71^ All these printing materials need to have characteristics such as printability, biocompatibility, degradation kinetics and byproducts, structural and mechanical properties, as well as material biomimicry.^72^

## 3 BIOPRINTING IN TISSUE ENGINEERING

### 3.1 Scaffold fabrication in bioprinting

In bioprinting, scaffold design plays an important role in determining the success of tissue regeneration.^73^ The architecture of the scaffold should fully mimic the native tissue structure to provide a framework for cell proliferation, nutrient diffusion, and tissue integration. Various design parameters, such as pore size, interconnectivity, and surface roughness, need to be considered comprehensively.^74^

First of all, the pore size of the scaffold will affect the behavior of cells such as adhesion and proliferation.^75^ Generally speaking, if the pore size of the scaffold is too small, it is difficult for cells to migrate through the scaffold. At the same time, it is vulnerable to distributing the nutrients needed by the cultured cells to individual cells. And, wastes produced by cells are not easily eliminated and may lead to cell death.^75^ Conversely, if the pore size of the scaffold is too large, the cells are easily dislodged, although the available surface area for cell adhesion is increased. Second, the size of the pore also affects tissue formation. Pore size needs to be in an appropriate range to maximize cellular function. For example, it has been found that pore sizes ranging from 100 to 300 µm are suitable for bone tissue engineering.^76^ In addition, the size of the aperture also affects the mechanical properties. There is a negative correlation between the pore size and the mechanical properties of the scaffolds, and too large a pore size will adversely affect the mechanical properties of the scaffolds.^77^ Meanwhile, when preparing bioscaffolds, there are requirements for their porosity. The higher the porosity, the higher the internal surface area to volume ratio of the scaffold the more favorable it is for cell attachment and bone growth.

Hierarchical designs^78^ can mimic the multiscale organization of tissues, providing mechanical strength and guiding cell behavior. With multilayer printing, it is possible to construct tissues or organs with complex three‐dimensional structures that more closely resemble the structure and function of natural tissues.^79^ Multilayer printing allows for the simultaneous printing of multiple layers of tissue, which greatly improves manufacturing efficiency and shortens manufacturing cycles compared with single‐layer printing. It is also possible to print layer by layer using different cell types or growth factors, thereby creating tissues or organs with multiple cell types that more closely resemble the composition and function of natural tissues.^80^ Moreover, multilayer printing allows the use of appropriate growth factors and nutrients as printing materials, thereby promoting cell growth and activity and improving the quality and function of the manufactured tissue or organ.^81^ What is more, multilayer printing can be personalized according to the patient's needs, thus better meeting the patient's needs.^82^

Gradients of biomaterials or bioactive factors^83^ can create spatial variations in the scaffold, promoting tissue development and organization. Specifically, a gradient of biomaterials or bioactive factors can provide different concentrations of nutrients, growth factors, cytokines, and so on to support cell growth and differentiation.^84^ This gradient design can enable cells to exhibit different growth and differentiation states in different regions, thus better mimicking the structure and function of natural tissues. In addition, gradients of biomaterials or bioactive factors can also regulate cell adhesion and migration behavior. Cells have different abilities to adhere and migrate in different regions, depending on the gradient of the biomaterial or bioactive factor.^85^ Such gradients can be designed to better control cell growth and differentiation, thereby better mimicking the structure and function of natural tissues. In conclusion, the gradient of biomaterials or bioactive factors plays an important role in bioprinted scaffolds, which can provide a suitable environment for cell growth and differentiation, and regulate the adhesion and migration behavior of cells, thus better mimicking the structure and function of natural tissues.

All the above factors affect the mechanical properties of the scaffold, which can be tailored by adjusting the composition, cross‐linking density, or porosity of the bioink. For example, increasing the concentration of polymer in the bioink can enhance scaffold stiffness, while adjusting the cross‐linking density can modulate the scaffold's elasticity.^86^ As a result, intricate scaffolds with desired architectures and mechanical properties offer unique advantages for tissue regeneration.

### 3.2 Integration of cell printing and bioprinting

Bioprinting techniques, as discussed earlier, involve the deposition of bioinks that contain cells within a biomaterial matrix, enabling the direct deposition of cell‐laden structures using bioprinting techniques. The cells within bioinks could maintain their viability and functionality during the printing process, allowing for the fabrication of tissues with desired cell distribution and organization.^87^ By printing layers of cells on special materials and positioning them precisely, specific tissue structures can be formed. Cellular bioprinting has many advantages, for example, cellular bioprinting can create tissues with complex three‐dimensional structures, whose structures and compositions can highly mimic those of natural tissues and thus more closely resemble actual physiological states.^88^ Cell bioprinting can also be personalized to meet the needs of the patient, such as printing patient‐matched organs or tissues for transplantation.^89^ With these complex structures, it is possible to shorten the cycle of drug development and testing. Of course, the use of cell bioprinting avoids the ethical issues associated with using intact animals for experiments.

The types of cells commonly used in cell printing depend on the fields of application and purposes. Here are some common cell types that are widely used in cell printing: (1) *Fibroblasts*: fibroblasts are a cell type widely found in human tissues and have a good ability to proliferate and differentiate. They can be used to build different types of tissues and organs. For example, researchers used the cells in conjunction with 4D laser printing to study deformation and reorganization during wound healing.^90^ (2) *Stem cells*: they have the ability to self‐renew and differentiate into many cell types. They are widely used in cell printing and disease treatment.^91^, ^92^ (3) *Stromal cells*: stromal cells are a class of pluripotent cells that are found in many tissues and play an important role in maintaining and repairing tissue function. They can be used to build the matrix structures that support tissues and organs. For example, laser‐assisted bioprinting of mesenchymal stromal cells promotes bone regeneration in a mouse model of cranial defects.^93^, ^94^ (4) *Cardiomyocytes*: cardiomyocytes are the main building blocks of heart muscle tissue and can be used to construct cardiac muscle tissue and artificial hearts.^95^, ^96^ (5) *Hepatocytes*: hepatocytes are the major cell type of the liver and have important metabolic and detoxification functions. Hepatocytes can be used to build liver tissues and bioreactors. Taymour et al.^97^, ^98^ first demonstrated alginate‐ and methylcellulose‐based bioinks for bioprinting of hepatocytes. (6) *Neurons*: neuron cells are the basic units that make up the nervous system, and they can be used to build neural tissue and simulate nerve conduction. Salaris et al.^99^, ^100^ reported a neural construct generated by 3D bioprinting of cortical neurons and glial precursors derived from human pluripotent stem cells.

However, there are some drawbacks and challenges associated with this technology:

The equipment and materials used for cell bioprinting are costly, resulting in an expensive overall manufacturing process.^101^ In addition, the cost of cells and growth factors required in the manufacturing process is also high, further increasing the manufacturing cost.^102^ And cell bioprinting technology requires precise control of cell growth and development, which is technically difficult. The manufactured tissue or organ needs to have a complex three‐dimensional structure, and the viability and functionality of the cells need to be ensured. During cell bioprinting, cells need to be separated from the donor and manipulated, which may cause damage or death to the cells.^103^ In addition, the printing process requires the use of highly viscous media, which may also cause damage to the cells.^104^ In terms of transplantation, cell bioprinted tissues or organs need to be addressed for immune rejection to avoid rejection in the patient. Immunosuppressive therapy is needed, but this increases the risk of infection and disease.^105^ At last, standards and quality control systems need to be established: cellular bioprinting technology needs to establish standards and quality control systems to ensure the quality and safety of the manufactured tissues and organs. This requires extensive experimentation and validation and the development of relevant regulations and standards.^106^ ^,^ ^107^

Although there are several challenges (Table 2), the integration of cell printing and bioprinting techniques holds great promise for advancing tissue engineering and regenerative medicine. With all these functional tissues, enhanced biological relevance can be created.

**TABLE 2:** Advantages and challenges of cell printing.

		Description	References
Advantages	Precise cell placement	Prints cells to a specific shape or trajectory	^108^
	Controlled cell organization	Enables the controlled organization of different cell type	^109^
	Cell–cell interactions	Facilitates cell–cell interactions and communication	^110^
	Multifunctional constructs	Incorporates multiple cell types, bioactive factors, and biomaterials	^111^
Challenges	Cell viability	Printing process of small cell sizes or delicate cell types	^112^
	Printing resolution	Deals with small cell sizes or delicate cell types	^113^
	Bioink compatibility	Combines with both the printing technique and the printed cells	^114^

## 4 BIOPRINTING IN DRUG SCREENING

### 4.1 Tissue models in bioprinting

Tissue models play a crucial role in promoting biomedical research, drug discovery, and personalized medicine. By combining bioprinting with advanced imaging techniques^115^ and CAD strategies, researchers can create in vitro tissue models with defined microenvironments, vascular networks, and cellular organization. These tissue models serve as potential tools for studying disease mechanisms, evaluating drug efficacy and toxicity, and developing patient‐specific treatments.^116^, ^117^, ^118^

**FIGURE 3:**
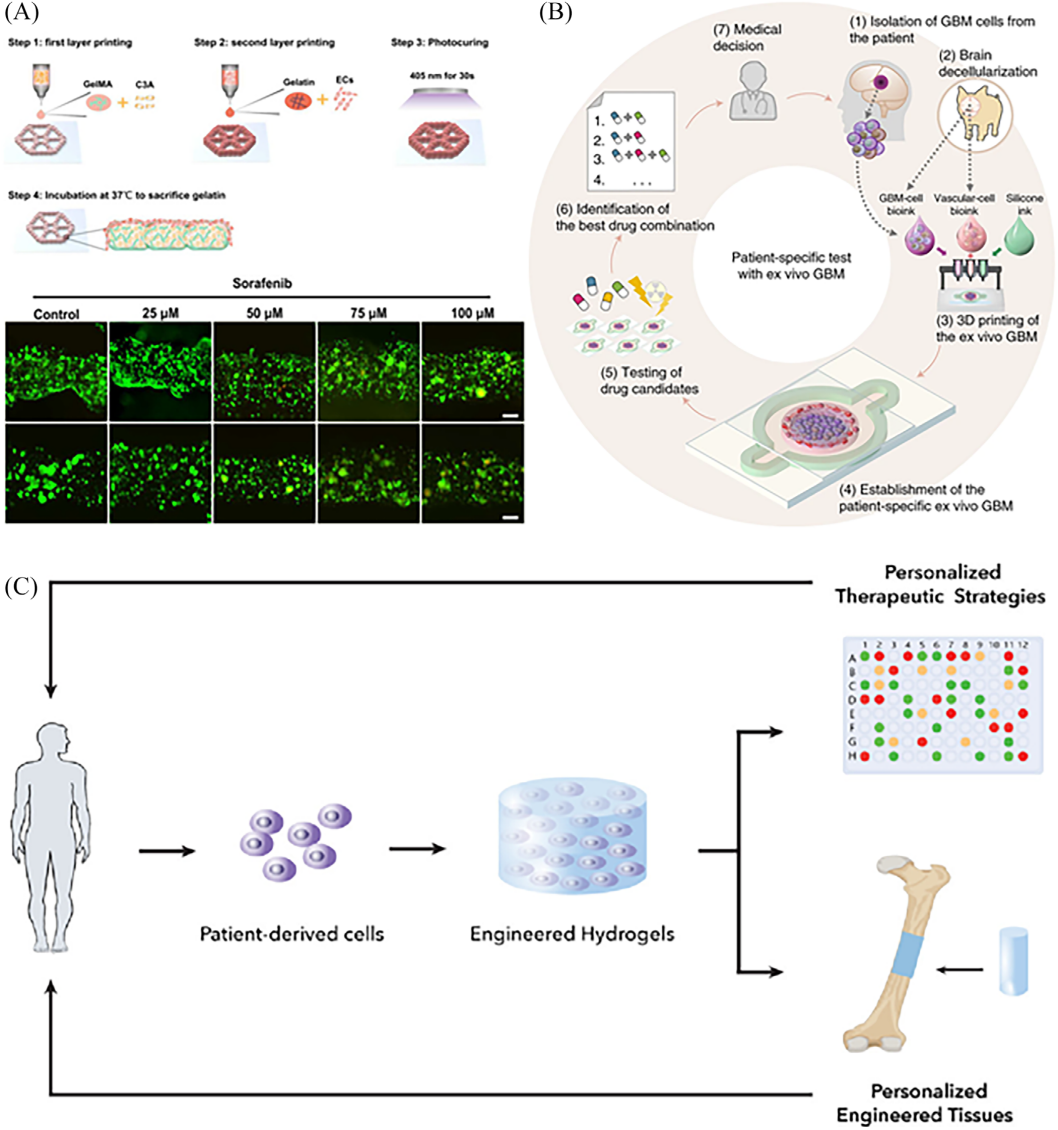
Bioprinting for drug screening. (A) Endothelialized liver lobule‐like constructs fabrication and drug evaluation.^201^ Copyright 2023 The Authors, published by MDPI. (B) Bioprinted organ‐on‐a‐chip for drug screening.^202^ Copyright 2019, Springer Nature. (C) Bioprinting creates artificial cell microenvironments for personalized tissue engineering and regenerative medicine applications.^132^ Copyright 2021, Elsevier.

One application of bioprinted tissue models is in the field of cancer research. For example, as shown in Figure 3A, endothelialized liver lobule‐like constructs have been fabricated and utilized for drug evaluation.^90^ Researchers have successfully explored many bioprinted tumor models that mimic the heterogeneity and cellular interactions observed in actual tumors.^110^, ^119^, ^120^ The structure and composition of these models can highly mimic natural tissues and are closer to the actual physiological state, which can better simulate the developmental process of cancer and the tumor microenvironment, and provide strong support for the study of cancer pathogenesis and drug screening.^121^, ^122^ Through bioprinting technology, a tumor model matching the patient can be manufactured, shortening the research and development cycle for personalized treatment and precision medicine. Such models can help doctors better understand the patient's condition and develop more precise treatment plans.^123^ Moreover, the use of bioprinting technology can avoid the ethical issues associated with the use of intact animals for experiments. Meanwhile, drug screening and testing through in vitro models can reduce risks and side effects to patients.^124^

Moreover, bioprinted tissue models have found applications in organ‐on‐a‐chip technology. For instance, bioprinted organ‐on‐a‐chip has been developed for drug screening (Figure 3B).^91^ These systems use microfluidic devices that replicate the functions and interactions of human organs.^125^ Bioprinting techniques can be combined with these devices, enabling the recapitulation of organ‐level functions and providing valuable insights into organ physiology, disease mechanisms, and drug responses.^126^ Organ‐on‐a‐chip can simulate real physiological environments and provide a more accurate in vivo model of the human body. By combining bioprinting with organ‐on‐a‐chip technology, the drug development process can be accelerated, the use of animal testing can be reduced, and the efficiency and accuracy of drug screening can be improved.^127^, ^128^ Bioprinting technology can be used to create patient‐matched cells and tissues, while organ‐on‐a‐chip can mimic a patient's organ function. This combination can enable individualized regenerative medicine to help patients regain function and improve treatment outcomes. The combination of these two technologies can optimize the use of resources and reduce manufacturing costs.^129^ By manufacturing tissues or organs outside the body, dependence on animal resources can be avoided, while reducing the need for raw materials and lowering production costs. By automating and digitizing the manufacturing process, more realistic and accurate tissues or organs can be manufactured quickly and accurately, improving production efficiency and quality, helping scientists better understand the physiological and pathological mechanisms of human organs, and advancing the progress and development of medical research.^130^, ^131^

Another area where bioprinted tissue models have shown significant potential is regenerative medicine. Bioprinting has successfully created artificial cell microenvironments for personalized tissue engineering and regenerative medicine applications (Figure 3C).^132^ By creating patient‐specific tissue constructs, researchers aim to overcome the limitations of traditional tissue transplantation and create tissue‐engineered building blocks with complex structures and specific functions, such as bone,^133^ cartilage,^134^ muscle,^135^ and skin,^136^ to promote tissue regeneration. The bioprinted scaffolds can be seeded with patient‐sourced cells to create personalized tissue implants that enhance the integration and functionality of regenerated tissues, thus solving the problem of organ donor shortages and avoiding possible rejection of allogeneic organ transplants.^137^

Therefore, the prospects and future of bioprinted tissue models are very broad and promising. As technology continues to develop, bioprinting technology will continue to be optimized and improved, making it possible to create more realistic and functional tissues and organs. However, the development of bioprinting technology also faces some challenges. For example, how to ensure that the printed tissues and organs survive and function properly in the body for a long period of time, and how to realize mass production and commercial application.^138^, ^139^ Consequently, continued research and exploration are needed in the future to overcome these challenges and promote the further development of bioprinting technology.

### 4.2 Bioprinting platforms for drug screening

Drug discovery and development are characterized by high costs, long lead times, and high risks. Despite the emergence of methods for simultaneously detecting protein and nucleic acid biomarkers,^140^ there is still a need for integration with other technologies. Bioprinting platforms have emerged as valuable tools for high‐throughput drug screening, enabling the fabrication of physiologically relevant tissue models for more accurate and efficient evaluation of drug efficacy and toxicity. These platforms combine bioprinting technology with advanced cell culture techniques and microfluidic systems to create functional organotypic models that closely mimic human tissues.^141^

One commonly used bioprinting platform for drug screening is the organ‐on‐a‐chip system.^142^ These devices integrate bioprinted tissue constructs with microfluidic channels, providing high throughput, efficiency, sensitivity, specificity, microvolume capacity, automation, and integration.^143^ This allows for the perfusion of media and nutrients to mimic the dynamic microenvironment of human organs in vitro. These platforms enable the study of drug responses in a more physiologically relevant context, providing deeper insights into tissue‐specific drug effects, metabolism, and toxicity at a lower cost and dependence on animal resources.

Another bioprinting platform for drug screening involves the use of multicellular spheroids or organoids which consist of multiple cells.^144^ They more closely resemble native features than 2D cell cultures do and have cell types and organizational structures similar to those found in original tissues. This degree of simulation makes organoids a powerful tool for studying human physiology and disease. Stem cells in organoids can self‐renew and maintain their phenotype, allowing organoids to remain stable over long periods of time.^145^ This property allows researchers to culture and study organoids for long periods of time. At the same time, organoids have strong plasticity and can be induced to differentiate into specific cell types as required by research. Additionally, the genome of organoids is relatively stable, allowing for a wide range of applications in drug screening,^146^ disease modeling,^147^ and other research needs while maintaining phenotypic and functional consistency. Bioprinting techniques enable the creation of spheroids or organoids with precise control over cell composition, spatial arrangement, and size.^148^ These 3D tissue‐like models accurately replicate the complexity of tissues and organs, providing a more accurate representation of drug responses. By incorporating multiple cell types and biomaterials, bioprinted spheroids or organoids can simulate the cellular interactions and heterogeneity observed in vivo, thereby enhancing the predictive value of drug screening assays.

Furthermore, integrating bioprinting platforms with advanced imaging and sensing technologies enhances capabilities for drug screening. Automated imaging systems capture images of printed tissue models at regular intervals to monitor tissue development, cell behavior, and drug responses over time. Advanced image analysis algorithms quantify various parameters such as cell viability, tissue morphology, and protein expression to provide quantitative data for drug screening evaluations.^149^ These integrated platforms offer valuable insights into drug pharmacokinetics, drug‐target interactions, and cellular responses that contribute to informed decision‐making in drug development.

In summary, bioprinting platforms offer significant advantages for drug screening, including the creation of physiologically related tissue models, scalability for high‐throughput screening, and integration with imaging and sensing technologies. These platforms have the potential to improve the efficiency and accuracy of drug discovery and development.

## 5 BIOPRINTING IN ORGAN REGENERATION

Bioprinting has shown immense potential in the fabrication of sophisticated organ structures, addressing the challenges associated with vasculature, intricate architectures, and heterogeneous cell populations. The successful bioprinting of functional organs requires careful consideration of various factors and advancements in technology. Advancements in bioprinting techniques have paved the way to regenerate organs for stimulating and reconstructing the function and structure of in vivo tissues and organs. The new achievements and emerging research directions in the field of bioprinting have shown promising prospects and potential applications.

### 5.1 Bioprinting of vascular networks

One of the primary challenges in bioprinting intricate organ structures lies in the integration of a functional vascular network. Blood vessels play a crucial role in delivering oxygen and nutrients to cells, as well as removing waste products from organs.^150^ The complex structure of blood vessels includes branches of varying diameters and curvatures, along with vessel segments serving different physiological functions. Bioprinted blood vessels necessitate the use of materials with excellent biocompatibility and functionality, alongside advanced manufacturing processes, to ensure that the printed blood vessels are compatible with human tissues and are able to perform normal physiological functions.^151^ The complexity of these structures and the unavailability of materials make it very difficult to create highly biomimetic blood vessels. After a single blood vessel is printed, it needs to be connected to other vessels to form a complete vascular network. This process requires a high degree of precision and skill to ensure the reliability and stability of the connectivity.^152^ Afterward, cells and tissues need to be integrated into the printed structure to realize the physiological function of the blood vessels. This process requires controlled cell growth and differentiation to ensure integration and functionality of the tissue.^152^ The layout and structure of functional vascular networks continue to be developed to meet the demands of specific and operational biological problems. Bioprinted blood vessels therefore face complex challenges that need to be overcome on multiple fronts. Recently, researchers have developed innovative strategies such as sacrificial bioinks^70^, ^71^ and biofabrication techniques^4^ to create perfusable vascular networks within bioprinted tissues. This enables the integration of intricate vasculature, mimicking the natural blood supply system.

### 5.2 Bioprinting of different cell types and biomaterials

In addition to vasculature printing, this technique has evolved to address the need for intricate architectures inside designed organs. To further develop automation and higher efficiency, some groups focused on the functional expansion of the biopringting organs using various cells and biomaterials. Advanced methods, such as multimaterial^37^ and multinozzle printing,^153^ allow for precise fabrication of different cell types and biomaterials, enabling the building of complex tissue structures. Scaffold‐free bioprinting approaches, such as spheroid‐based bioprinting, have also been invented to achieve self‐assembled cellular structures and improve cellular organization within created organs.^154^

Furthermore, bioprinting aims to replicate the heterogeneous cell populations that emerged in natural organs. This involves the careful selection and incorporation of multiple cell types with distinct functionalities, ensuring the presence of parenchymal cells, stromal cells, and supporting cells.^155^ Some relevant techniques, such as cell‐by‐cell deposition or simultaneous coprinting of multiple cell types, enable the precise placement and subtle arrangement of different cell populations, contributing to the development of functional and physiological organ models.^4^

### 5.3 Bioprinting of complex structures

In the above studies, the researchers focused on how to integrate different types of cells and biomaterials. Other researchers have evaluated the quality and complexity of bioprinting structures by mimicking the state of in vivo tissues and organs. The significance of constructing complex structures in bioprinting is that they can mimic the structure and function of real tissues and provide more realistic and accurate models for medical research,^156^ drug development^157^ and regenerative medicine.^158^ Specifically, the significance of constructing complex structures is as follows:

*Simulating real tissues* ^159^: through bioprinting technology, complex structures that are highly similar to human tissues can be created. These structures are not only morphologically similar to real tissues, but also functionally mimic the physiological activities of real tissues as closely as possible. This allows scientists to better simulate the growth, development and physiological functions of human tissues in the laboratory, and thus study human physiology and disease mechanisms more accurately.

*Drug development and testing* ^160^: complex structures can be used for drug development and testing. By creating models in vitro that are highly similar to human organs, scientists can evaluate the effectiveness and safety of new drugs, greatly shortening the drug development cycle and reducing costs. At the same time, these models can also be used to test the efficacy and side effects of different drugs on specific tissues, providing a stronger basis for clinical trials.

*Regenerative medicine* ^161^, ^162^: complex structures can provide better solutions for regenerative medicine. Through bioprinting technology, organs or tissues matching the patient can be created for transplantation and repair. This can not only solve the problem of organ donor shortage, but also avoid the possible rejection of allogeneic organ transplantation. At the same time, these complex structures can also be used to study the mechanisms of organ regeneration and repair, providing new ideas and methods for treating organ failure and diseases.

*Promote interdisciplinary cooperation* ^163^: bioprinting complex structures requires cross‐disciplinary cooperation, including biology, materials science, biomedical engineering, and so on. Such interdisciplinary cooperation can promote communication and cooperation between different fields and promote the development and innovation of the discipline.

*Personalized treatment* ^164^: through bioprinting technology, organ models can be manufactured to match the patient's genetic information and physiological characteristics. This kind of personalized treatment can provide patients with more precise treatment plans, improve treatment effects, and reduce side effects.

### 5.4 Bioprinting for organ regeneration

Living organs are always performing complex biochemical and biophysical processes to maintain their physiological functions. In this case, only one type of organ is not enough. To mimic the function of in vivo multiorgan systems, bioprinting and integrating of multiorgans were designed for various purposes.^165^ The development of specific bioprinting platforms and techniques is essential for versatile organ regeneration. Various factors, including cell source, vascularization, and functional integration, need to be considered in the design of bioprinting systems for organ regeneration.^166^, ^167^

Functional integration of bioprinted tissues with the host organism is an important consideration. Bioprinting platforms are being designed to enable the integration of bioprinted organs with the existing tissue through techniques like surgical implantation, tissue fusion, or biofabrication of interface layers.^168^, ^169^ This promotes the seamless integration of bioprinted organs into the host, facilitating their functionality and long‐term survival. In vivo bioprinting introduces the concept of additive manufacturing into the clinical settings for tissue injury treatment. Nowadays, handheld and multiaxis bioprinting devices are well‐established, and minimally invasive devices are emerging approaches.^170^ This method deposits bioink directly into the defect site in the patient's body, effectively solving the problem of fabricating and implanting irregularly shaped scaffolds and enabling rapid on‐site management of tissue damage. Another clinical application is artificial intelligence and robotics.^171^ It is equipped with a highly free‐flowing soft print head integrated into a flexible robotic arm that can deliver multilayered biomaterials to internal organs/tissues. In addition, bioprinting in space will be the next frontier in tissue engineering. Baio et al.^172^ cultured neonatal and adult human cardiovascular progenitor cells on the International Space Station and investigated changes in the expression of microRNAs and genes related to mechanotransduction, cardiogenesis, cell cycling, DNA repair, and paracrine signaling.

In a word, the development of 3D bioprinting technology plays an important role in organ growth and the emergence of innovative biomaterials. This technology will help to replace human organs, as in some cases they outperform vital organs. However, there are still difficulties in realizing the printing of living tissues, which are still only in the research and development stage, although there are already teams that have developed the printing of living cells, and tissues such as nerves and blood vessels can already be 3D printed, but this type of application will take a long time if it is really going to be operated in an industrialized way.

## 6 BIOPRINTING IN BIOSENSORS

### 6.1 Sensor structures in bioprinting

**FIGURE 4:**
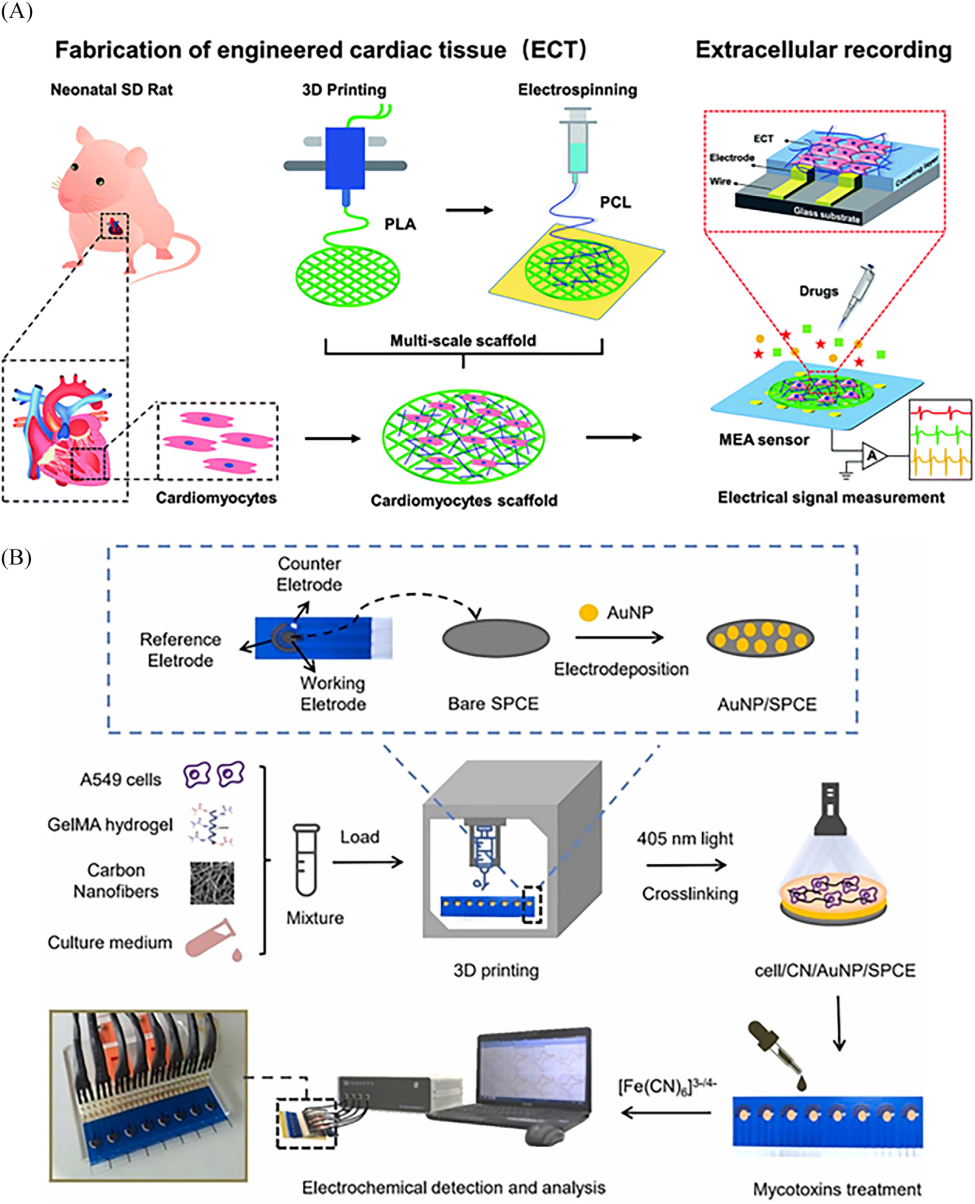
(A) Biosensor of bionic engineered cardiac tissue based on a 3D‐printed porous scaffold and microelectrode arrays.^203^ Copyright 2019, RSC Pub. (B) Schematic representation of the route for the preparation of the 3D‐printed electrochemical cell‐based biosensor.^186^ Copyright 2023, Elsevier.

Sensing elements including cells, enzymes, nanoparticles, or other biomolecules, allow for the development of multiple types of biosensors. For example, a bionic biosensor using bioengineered cardiac tissue as a sensitive element has been developed based on a 3D printed porous scaffold and microelectrode arrays (Figure 4A).^111^ Bioprinting enables the spatial organization of these components within a biocompatible scaffold, creating sensor structures that closely mimic the complexity and functionality of natural tissues (Table 3).

**TABLE 3:** Parameters comparison of biosensors.

Performances	Cell‐based biosensors	Enzyme‐based biosensors	Nanoparticle‐based biosensors
Sensitivity	√	√	√
Selectivity	√	√	√
Stability	Insufficient	√	×
Biocompatibility	√	√	×
Detection mode	Multiple	Multiple	Multiple
Longevity	Limited	Limited	Days to months
Responsibility	√	√	√
Diversity	√	√	√
Difficulty of production	Complicated	Easy	Complicated
Security	√	√	×
Repeatable	×	×	×
Impact of the environment	√	√	√

√ denotes the corresponding performance is good and × denotes the corresponding performance is not available.

By incorporating living cells into the sensor structures, bioprinting enables the development of cell‐based biosensors that can respond to specific biological signals or detect target analytes with high sensitivity, which can be used for real‐time monitoring of physiological parameters, disease diagnostics, and drug screening in the field of biomedical sensing.^173^ Figure 4B is a schematic representation of the route for the preparation of the 3D printed electrochemical cell‐based biosensor.^112^ Simple to operate and easy to integrate into cell culture systems, cell‐based biosensors allow real‐time monitoring of indicators of cell activity, cellular metabolites, and intracellular molecules. It is a noninvasive monitoring method that does not cause damage to cells or interfere with their normal physiological functions. It can also be designed and optimized for different cell types and physiological indicators, with a wide range of applications and versatility.

Additionally, enzymes are potential modules that can catalyze specific biochemical reactions, enabling the detection and quantification of target molecules.^174^ Enzyme‐based biosensors utilize the catalytic action of enzymes and are capable of detecting target substances with high sensitivity. Enzymes are highly selective and specific to the target substance and can detect low concentrations of the target substance. The enzyme is a biologically active substance with a certain degree of stability. During the preparation and use of the enzyme sensor, the activity of the enzyme can be maintained for a longer period of time, thus improving the stability and reliability of the sensor.^175^ It can be integrated with other sensors or circuits to form a multifunctional and intelligent detection system. This helps to improve the integration and intelligence of the sensor system. The enzyme sensor has a wide range of applications in the fields of biomedicine,^176^ environmental monitoring,^177^ and food safety.^178^ It can be used to detect target substances such as biomolecules, organics, inorganics, and so on, providing a convenient and efficient means of detection in various fields. Bioprinting techniques optimize the interaction of enzymes with target analytes and improve the sensor's sensitivity and selectivity.

Furthermore, nanotechnology also plays a very important role in the field of sensing.^179^ Nanoparticles serve as signal transducers, amplifying the detection signals and improving the sensor's detection limits. By controlled positioning of nanoparticles, it facilitates the interaction of sensors with target analytes and enhances the overall sensor performance. Nanoparticles have a very high surface area to volume ratio, small size, and short diffusion paths, which allows them to respond quickly to changes in the outside world, thus shortening the response time and improving the response speed and sensitivity of the sensor. With rich physical and chemical properties, good thermal and chemical stability, and high surface activity, a variety of sensing functions can be realized through the design and modification of the material to improve the stability and life of the sensor.^180^, ^181^, ^182^ Moreover, nanomaterials can be synthesized in various ways, and the cost can be reduced by mass production.^183^, ^184^, ^185^

### 6.2 Bioprinting platforms for biosensors

The exploration of specialized bioprinting platforms tailored for biosensor applications sheds light on achieving optimal sensor performance and functionality. This section discusses the integration of sensing components, signal transduction elements, and microfluidic systems within bioprinting platforms.

To create biosensors using bioprinting platforms, it is necessary to precisely deposit and position sensing elements, such as cells, enzymes, or nanoparticles, in specific locations within the sensor structure.^186^ This requires bioprinting techniques that process high resolution and accuracy, allowing for the controlled targeting of sensing elements with sub‐millimeter or even sub‐micrometer precision. In 3D printing biosensors, the issues that need to be paid attention to when printing different biomolecules mainly include the following aspects:

*Activity protection of biomolecules* ^187^: during the printing process, biomolecules may be affected by mechanical force, heat, chemical reagents, and so on, resulting in the reduction or inactivation of their activity. Therefore, measures need to be taken to protect the activity of biomolecules, such as controlling the printing temperature and optimizing the printing parameters.

*Compatibility of printing materials* ^188^: different biomolecules require the use of different printing materials to ensure that the printed sensor devices do not poison the biomolecules or affect their activity. Thus, it is necessary to choose the appropriate printing material according to the nature of the biomolecule.

*Surface treatment of the printing substrate* ^189^: the surface treatment of the printing substrate has an important impact on the immobilization of the biomolecules and the performance of the sensor. Suitable surface treatment, such as surface coating, chemical modification, and so on, needs to be selected to improve the binding of biomolecules to the substrate.

*Batch consistency* ^190^: for sensors manufactured in batches, the consistency of biomolecule quality and performance in each batch needs to be ensured to ensure the stability and reliability of sensor performance.

*Cross‐contamination issues* ^191^: when printing different types of biomolecules in succession, care needs to be taken to prevent cross‐contamination between different types of molecules so as not to affect the performance of the sensor.

*Environmental factors*: environmental factors such as temperature, humidity, and light need to be controlled during the printing process to ensure the stability and activity of the biomolecules.

In addition to sensing components, detected signals need to be converted into measurable outputs by transduction elements of biosensors, such as electrodes or optical components.^186^, ^192^ Integration of transduction elements within the bioprinted scaffold ensures efficient signal capture and measurement. There are various types of transducers for biosensors, and the common ones include electrochemical transducers, optical transducers, and thermal transducers.^193^ Among them, electrochemical transducers include electrochemical electrodes, field effect tubes, and so on; optical transducers include photoconverters, photosensitive tubes, and so on; and thermal transducers include thermistors, thermocouples, and so on. These transducers can convert the reaction between biomolecules into measurable electrical,^194^ optical^195^ or thermal signals,^196^ and so on for quantitative detection and analysis of biomolecules. When selecting a transducer, factors such as its sensitivity, stability, reproducibility, immunity to interference, and compatibility with biomolecules need to be considered.

Furthermore, microfluidic systems also play an emerging role in biosensing applications by enabling the controlled transport and manipulation of samples and reagents.^197^ Bioprinting platforms could incorporate microfluidic channels, chambers, or valves, allowing for precise sample handling, real‐time dynamic observation, and controlled fluid flow within the biosensor device, which enhances the performance and functionality of bioprinted biosensors.^198^

## 7 CHALLENGES AND FUTURE PERSPECTIVES OF BIOPRINTING

In recent years, the degree of population aging has deepened, the elderly population's osteoporosis, femur and vertebrae fractures, tooth wear and tear, cardiovascular and cerebral vascular diseases, tumors, and other diseases’ incidence rate are rising, accelerating the process of population aging and providing a broad space for demand in the development of the 3D bioprinting market. Currently, under the background of the accelerating aging process and expanding application fields of 3D bioprinting technology, its market scale is expanding continuously. From the market pattern, the current 3D bioprinting companies’ focus on the field has not been unified. Although some bioprinted bone tissue products (such as CaOSiO_2_–P_2_O_5_–B_2_O_3_ glass–ceramic implants) have also entered clinical trials for the clinical repair of fractures, bone defects, and other bone‐related problems,^199^ the 3D bioprinting market pattern is relatively fragmented, and the industry's future development still has huge space. At the same time, the development of the 3D bioprinting industry is still facing the difficulties of technology, costs are higher, the key material bio‐ink categories are fewer, the standard system has not been perfected, the transformation of scientific research results is difficult, and other dilemmas, and the industry development is still facing a huge challenge. At present, the development of the 3D bioprinting industry is facing both opportunities and challenges, and the future development of the industry needs to seize the opportunities and break through the challenges to promote the continuous development of the industry.

With the development of 3D bioprinting and further cross‐fertilization of disciplines, it has the potential to produce disruptive breakthroughs in the field of in vitro life systems engineering. From the perspective of technology development trends, 3D bioprinting technology lays the scientific foundation for the expansion and extension of manufacturing science from using single structural materials to using functional materials, biomaterials, and life materials disciplines; the development of stem cell technology and bio/life materials provides the necessary basic materials; cellular 3D printing provides the core means of manufacturing (printing of advanced biological models, coding biological models, etc.); and the integration of micro–nano technology and microfluidic chip technology can be integrated to manufacture advanced biomimetic bioreactors for cultivating life systems and life mechanical devices. Based on cell 3D printing technology, the construction of large functional tissues and organs such as the heart, liver, pancreas, uterus, lungs, and so on using cells and bioactive materials such as embryonic stem cells, induced pluripotent stem cells, novel bioinks, and so on, is the frontier and hotspot of current research. This technology brings new opportunities for biofabrication of complex tissue structures to mimic pathological microenvironments. In the future, it has the potential to bring a disruptive impact to the fields of regenerative medicine, tumor therapy research, and new drug development.

In vitro 3D tissue/organ coding models and in vitro micro physiological systems are an emerging research concept and direction to better improve the accuracy of drug testing and shorten the drug development cycle. The technology is based on 3D printing technology, microfabrication technology, and so on, and utilizes bio‐microfluidics to simulate the activities and physiological properties of organs on a chip. Microfluidic technology has realized in vitro simulation of the heart, liver, lung, and other systems to varying degrees. Organ chips and humanoid chips could fundamentally change the means of drug detection and bring disruptive changes to the development of new drugs, becoming a new means of researching and treating cancer, tumors, and other diseases. It can be seen that in vitro micro physiological systems could more realistically simulate the in vivo environment and become an effective alternative to animal experiments in the near future.

## AUTHOR CONTRIBUTIONS

Shuge Liu, Liping Du, and Chunsheng Wu proposed the idea together. Shuge Liu, Yating Chen, Yushuo Tan, and Zhan Qu performed main text writing and production of some figures and tables. Zhiyao Wang, Minggao Liu, and Yundi Zhao contributed with the introduction writing and full text modification. Liping Du, Chunsheng Wu, and Yushuo Tan contributed with funding acquisition and supervision. Zhan Qu, Yushuo Tan, and Liping Du performed the adjusting article format and language checking. Chunsheng Wu contributed with the enhancement of main text writing, production of some figures and tables, and payment for publishing fee. All the authors have read and approved the final manuscript.

## CONFLICT OF INTEREST STATEMENT

No potential conflict of interest was disclosed.

## ETHICS STATEMENT

Not applicable.

## Data Availability

Not applicable.
